# Active Video Games Using Virtual Reality Influence Cognitive Performance in Sedentary Female University Students: A Randomized Clinical Trial

**DOI:** 10.3390/life14121651

**Published:** 2024-12-12

**Authors:** Mshari Alghadier, Taif Alharbi, Nada Almasoud, Abdulaziz A. Alshalawi

**Affiliations:** 1 Department of Health and Rehabilitation Sciences, Prince Sattam bin Abdulaziz University, Alkharj 11942, Saudi Arabia; taifmajedalharbi225@hotmail.com; 2 Department of Physical Therapy, Maternity and Children’s Hospital in Alkharj, Alkharj 16278, Saudi Arabia; pt_nada@hotmail.com; 3 Department of Family Medicine, Ad Dirryah Hospital, Riyadh 13717, Saudi Arabia; abaalshalawi@moh.gov.sa

**Keywords:** physical activity, virtual reality, active video game, cognitive performance, MoCA

## Abstract

Background: Virtual reality (VR) is an emerging technology that is proving to be effective in encouraging physical activity (PA) and improving health. Although regular PA has many advantages, physical inactivity continues to be a significant global health concern. Using an ActivPAL for PA assessment, this study examines the effects of an active video game (AVG) using VR on cognitive function among female university students. Methods: We randomly divided 44 sedentary female university students (mean age 21.3 years, SD 1.12 years) into two groups, the control group and VR group. During the study period, the VR group was required to play the Beat Saber VR game for 20 min, while the control group was required to remain quiet. Their cognitive performance was evaluated using the Montreal Cognitive Assessment (MoCA)—Arabic version pre- and post-test, and the PA level and intensity were tracked using the ActivPAL. Results: There was a significant difference between the MoCA total score pre-test (mean = 22.3, SD = 2.25) and the MoCA total score post-test (mean = 23.4, SD = 2.48), t (23) = 1.87, *p* = 0.03. The VR game significantly influenced the naming, abstraction, and orientation components of the MoCA scale (all *p* ≤ 0.05). The intensity of PA generated by the VR game was equivalent to moderate-to-vigorous PA, with a mean of 4.98 metabolic equivalents of task (MET) (SD = 1.20). Conclusions: The VR game improved the cognitive ability compared to the control group, suggesting that VR games have a positive impact on cognitive function. Physically inactive female university students have been found to benefit from VR games in terms of their cognitive function.

## 1. Introduction

Physical activity (PA) is defined as “any movement produced by skeletal muscle contractions that increases energy expenditure above the basal level” [1]. It is necessary for the maintenance of mental, emotional, and physical health, as well as in strengthening bones and muscles and maintaining a healthy weight [2,3,4]. Regular PA reduces the risk of noncommunicable diseases such as cardiovascular disease, diabetes, and major causes of mortality and morbidity. Despite the numerous benefits of regular PA, physical inactivity and low fitness levels remain major problems in global health [5]. Worldwide, it is ranked as the fourth most important risk factor for mortality, accounting for 6% of all deaths and affecting approximately 31% of all adults [6,7]. As our society’s physical, social, and economic environments have changed, this behavior has become more prevalent in recent years, posing a serious threat to public health [8]. Furthermore, ageing and a lack of time seem to be the most frequently stated barriers to PA in developed countries. Income may also influence physical inactivity, since lower-income individuals are less likely to meet the PA recommendations than higher-income individuals [9].

University students are more likely to develop sedentary behavior due to the amount of time that they spend in classrooms, studying, or in front of computers [10]. As reported by the American College Health Association [11], only 38% of college and university students (41% of males and 37% of females) meet the recommendations of the American College of Sport Medicine [12] regarding moderate-intensity exercise (at least 30 min five days per week) and vigorous-intensity exercise (at least 20 min three days per week). Moreover, 27% of the respondents reported that they did not participate in moderate exercise, while 48% reported engaging in no vigorous exercise. Another study revealed that 49% of students engaged in PA regularly, while the remainder did not [13]. Researchers have identified time constraints, busy academic schedules, laziness, a lack of training partners, inadequate facilities, a lack of confidence, and a lack of support as the most reported barriers to PA in college students [14,15].

In Saudi Arabia, there is a higher level of PA among males than among females [16,17,18,19,20,21]. This may be explained by the fact that females face social pressures in many traditional communities, which have historically associated physical strength and athleticism with maleness and viewed femininity as incompatible with vigorous activity. It was found that more than half of the students at health colleges in Saudi Arabia were physically inactive, while only 12% were highly active [22]. Additionally, Al Salim (2023) found that 41.8% of Saudi college students had a moderate level of PA, with female students experiencing greater barriers to being physically active than their male counterparts [23]. In addition to the lack of a suitable place and a lack of exercise partners, Khalaf et al. (2013) found time constraints to be the primary cause of irregular exercise among female students [24].

During the past few decades, computer usage has shifted away from stationary to mobile due to the popularity of technologies such as virtual reality (VR) [25]. VR refers to a physical environment that is created artificially with the aid of technology, consisting of a series of manipulated events, including visual, auditory, and other perceptual inputs, intended to be experienced by the individual [26]. There may be non-immersive VR environments as well as fully immersive environments that utilize head-mounted displays (HMDs). This emerging technology is highly effective and attractive in promoting PA and health [27].

Among the applications of VR that foster PA is exergaming or active video games (AVGs), which are defined as “video games that require bodily movement to play and function” [28]. Recently, exercise gaming has received a great deal of attention due to the development of commercial systems (such as the Nintendo Wii, PlayStation EyeToy, and Microsoft Kinect) [29,30,31]. It applies to a variety of settings and contexts (e.g., hospitals and schools), as well as to different age groups and goals [32]. Considering that exergames can induce exertion, they may have similar effects to traditional exercise, but with greater motivation, enjoyment, and engagement [33]. According to a 12-week randomized controlled trial (RCT) that examined the relationship between exergames and quality of life in healthy young adults, playing exergames significantly improved physical function, general health, and social functioning [34]. Exergames, played for 12 consecutive weeks, significantly improved physical fitness in college students, including cardiovascular health, muscle strength, and the three-minute step test results [35]. Additionally, research has shown that a fully immersive VR setting enhances the player’s game experience, immersion, positive effects, empathy, and physiological and psychological outcomes in comparison with non-VR settings [27,36,37]. A meta-analysis of 17 studies found that exergames improved the cognitive performance in clinical and non-clinical populations when compared to control groups. Exergames have been shown to be effective in enhancing executive functions, including inhibition, flexibility, attention, processing speeds, and visuospatial abilities [38].

An executive function is a higher-level cognitive process that is capable of managing and directing more basic functions effectively, thereby allowing for purposeful and controlled behavior. Intact executive functions are crucial for optimal mental and physical health, academic success, daily functioning, and overall growth. They are composed of three fundamental executive functions (inhibition, working memory, and cognitive flexibility). The first, inhibition, involves the ability to regulate one’s thoughts, attention, emotions, and actions; the second, working memory, involves holding information in mind and mentally working with it; and the third, cognitive flexibility, involves the capacity to switch between tasks, shift perspectives, and adapt to changing environmental demands [39]. Different standardized tools are used to assess cognitive ability, including the Wisconsin Card Sorting Task (WCST) [40], Montreal Cognitive Assessment (MoCA) [41], Mini-Mental Status Exam (MMSE) [42], Modified Mini-Mental State Examination (3MS) [43], and Mnemonic Similarity Test (MST) [44]. An individual’s cognitive abilities are crucial in maintaining optimal mental and physical health, academic success, day-to-day functioning, and overall growth [45]. In several studies, PA has been shown to influence the morphology and function of different brain areas in both humans and animals, thereby improving general cognition and cognitive performance [46,47,48]. According to the findings, young adults can significantly improve their cognitive abilities by increasing their PA levels [49].

Therefore, the purpose of this study was to explore the impact of AVGs using VR on the cognitive function of sedentary female university students using an objective PA measure. We hypothesized that the cognitive test scores of the VR group would improve after the VR game session compared to the control group. The AVG using VR was expected to increase the PA levels and therefore influence the cognitive abilities of sedentary female university students.

## 2. Methods

### 2.1. Participants and Study Design

Participants were invited to this RCT through advertisements sent via email and posters displayed in common student areas at Prince Sattam bin Abdulaziz University—Female Campus. The sample size was determined using the convenience sampling method. To answer the intended research question, the gender was limited to female students only; this also served to enhance the study’s internal validity. To control for potential confounding variables, strict inclusion and exclusion criteria were applied. The inclusion criteria were an age between 18 and 23 years, female college students, low PA levels (total weekly reported MET minutes ≤ 1500 min/week) calculated using the International Physical Activity Questionnaire—Short Form (IPAQ-SF) [50], no previous medical condition affecting their PA levels, no visual impairments, and no experience with the VR game used in the trial. This trial was registered at ClinicalTrials.gov (Identifier: NCT06646874).

**Figure 1:**
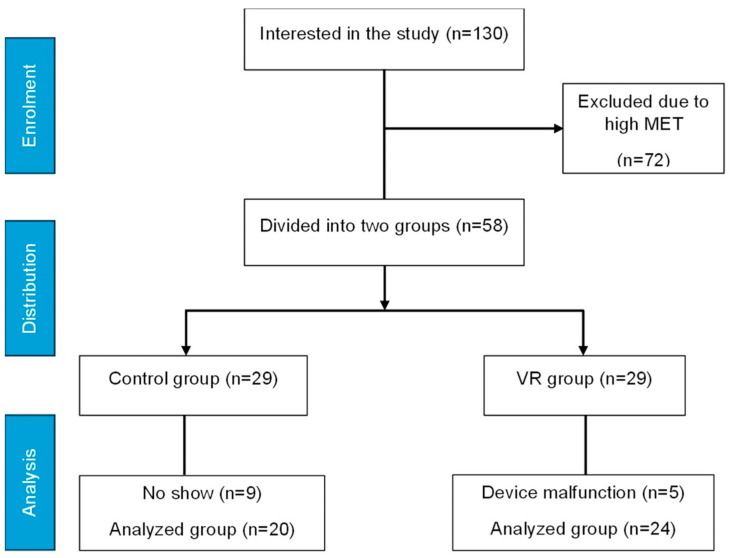
Study recruitment and distribution flow chart.

A total of 130 female students showed interest in the study by completing the pre-screening questionnaire using the IPAQ-SF. The majority of these students were excluded due to high PA levels, as identified by the IPAQ-SF (weekly reported MET > 1500 min/week). Fifty-eight participants met our inclusion criteria and were asked to attend the first visit. Ineligible participants were contacted through email and thanked for their interest in the study. Furthermore, five participants were excluded from the final analysis due to device misplacement or malfunction, and nine participants did not attend the first visit, leaving 44 eligible participants, as shown in Figure 1.

### 2.2. Procedure

The experiment was conducted at the Human Biomechanics Laboratory of the College of Applied Medical Sciences of Prince Sattam bin Abdulaziz University between February 2024 and May 2024. During the briefing, all questions were answered to ensure that the participants understood the experiment and its objectives. On the day of the experiment, demographic information was collected, including age, height, weight, body mass index (BMI), and preferred leg. An eye-level weight beam scale (Detecto, Webb City, MO, USA) was used to determine the weight and height of the subjects. To identify the preferred leg, the participants were observed ascending stairs or kicking a ball.

The participants were recruited and assigned to one of two groups, either the control group or the VR group. A simple randomization technique was used to assign the participants to the groups, using the random list function in Microsoft Excel (Microsoft Corp., Redmond, WA, USA). A standardized inclusion criterion in terms of age, gender, physical activity level, and health condition minimized selection bias. The VR group was required to play a 20 min game using Beat Saber (Beat Games, Prague, Czech Republic) VR in one session, while the control group was asked to sit quietly for 20 min without any activities assigned in one session. This study used an accelerometer from ActivPAL (PAL Technologies Ltd, Glasgow, UK) to capture the PA levels of the VR group throughout the VR game. Following previously published validation studies and manufacturer recommendations, a strip of Tegaderm (3M, St. Paul, MN, USA) was attached to the preferred leg’s front thigh [51,52].

Each participant in the VR group completed a five-minute familiarization session with the VR game before the experimental session. Following the familiarization session, the participants in the VR group were instructed to complete the Montreal Cognitive Assessment (MoCA)—Arabic version (pre-test: Arabic version 8.1 and post-test: Arabic version 8.2) [53]. The control group completed the MoCA pre- and post-tests 20 min apart. A debriefing was conducted for the participants following the conclusion of the experiment.

#### 2.2.1. Montreal Cognitive Assessment (MoCA)

Developed as a screening instrument to detect mild cognitive impairment, the Montreal Cognitive Assessment (MoCA) uses a series of questions to assess cognitive impairment [41]. A paper-and-pencil assessment is administered and scored out of 30 points. In addition to attention, concentration, executive functions, memory, language, visuospatial skills, abstraction, calculation, and orientation, the MoCA assesses several cognitive domains. There are 56 language and dialect translations of the MoCA, and it is widely used throughout the world. A copy of the test and instructions may be obtained for free from the MoCA’s official website at http://www.mocatest.org (accessed on 14 January 2024), and no permission is required for clinical or educational use. This experiment used the MoCA Arabic versions 8.1 and 8.2, which have been validated and tested in a variety of contexts [54,55,56].

#### 2.2.2. International Physical Activity Questionnaire (IPAQ)

An international consensus group developed the International Physical Activity Questionnaire (IPAQ) in 1998 for young to middle-aged adults [57,58,59]. With two versions available, the IPAQ is known as the most widely used PA questionnaire, with 31 items in the long form (IPAQ-LF) and 9 items in the short form (IPAQ-SF) [60]. In the IPAQ-SF, four levels of intensity are measured: (1) vigorous-intensity activity such as aerobics, (2) moderate-intensity activity such as leisure cycling, (3) walking, and (4) sitting. Both versions have recently been validated in Arabic-speaking countries for a variety of populations [61].

#### 2.2.3. ActivPAL

The ActivPAL (PAL Technologies Ltd, Glasgow, UK) is a posture and activity tracker that uses a tri-axial accelerometer to measure movement and activity. The device collects information such as bouts of sitting, lying, standing, and stepping and allows data to be retrieved by week, day, or hour [62]. As a valid and reliable tool to detect PA, the ActivPAL can be used in a variety of populations and settings [63]. The ActivPAL was calibrated according to the manufacturer’s guidelines and using a standardized protocol. Each participant was instructed to wear the ActivPAL device on the midline of the thigh and fix it in place with adhesive tape. To manage the ActivPAL and visualize and process the data, the PALanalysis software (V.7.2.32, PAL Technologies, Glascow, UK) was downloaded by registering as a user on the PAL website. Instructions on device programing and downloading the data were followed through the manual provided by the manufacturer. From the ActivPAL, the following variables were collected for each participant in the VR group: total MET, total number of steps, total time stepping, number of sit-to-stands, and number of stand-to-sits. The PA intensity was categorized as sedentary activity (<1.5 METs), light activity (1.5–2.99 METs), or moderate-to-vigorous PA (MVPA) (≥3 METs) [64].

#### 2.2.4. Virtual Reality Game and Headset

This study used a Meta Quest 2 VR headset (Meta Platforms, Inc., Menlo Park, CA, USA) and a Razer Blade 15 gaming laptop (Razer Inc., San Diego, CA, USA) equipped with an Intel Core i7-10750H processor, 16GB RAM, 513GB SSD, a GeForce RTX 2070 graphics coprocessor, and a 15.6-inch 4K OLED screen. The Meta Quest 2 features a per-eye LCD display with a resolution of 1832 by 1920, a display rate of up to 90 Hz, four infrared cameras, and integrated speakers [65]. For 20 min, the participants played Beat Saber games with different levels of difficulty according to the game classification (one song with low-level difficulty, one song with moderate-level difficulty, and two songs with hard-level difficulty) [66]. Beat Saber is an interactive game that requires the player to move or bend their body to cut through blocks moving in their direction or avoid larger ones by stepping to the side.

### 2.3. Statistical Analysis

Statistical analyses were carried out to determine the significance of any observed differences between the groups. Descriptive statistics were used to characterize the sample; frequencies and percentages were reported for categorical variables, while the mean and standard deviation (SD) were reported for continuous variables. Using Kolmogorov–Smirnov tests and graphical inspection methods, including histograms and normal quantile-quantile charts, all continuous variables were examined for normality. An independent-sample *t*-test and paired-sample *t*-tests were performed to determine the differences in age, height, weight, BMI, PA level, and cognitive ability between the groups. The interpretation of Cohen’s d was as follows: 0.2 = small effect, 0.5 = moderate effect, 0.8 = large effect. Each statistical test was carried out with significance set at *p* < 0.05, and the analyses were performed using R version 4.0.3 (10 October 2020).

### 2.4. Ethical Considerations

Written informed consent from the participants was acquired prior to data collection, and a participant information sheet was given to each participant. The Declaration of Helsinki’s ethical guidelines were followed when conducting this experiment. This study was approved by the Departmental Ethical Committee, Health and Rehabilitation Department, Prince Sattam bin Abdulaziz University, Saudi Arabia (No. RHPT/023/007; Date: 1 August 2023). Throughout this study, the participants’ data confidentiality and anonymity were guaranteed. As an incentive to facilitate participation, the participants were included in a draw to win one of six gift cards (SAR 100 each).

## 3. Results

A total of 44 female students with a mean age of 21.3 years (SD = 1.12), weight of 57.2 kg (SD = 11.8), and height of 158 cm (SD = 5.35) were recruited in this study. Among the sampled individuals, the average BMI was 22.9 kg/m^2^, a value within the normal range of body weights. The study sample was divided into a control group (n = 20) and VR group (n = 24). All participants were identified as having a low PA level according to the IPAQ-SF weekly MET score (MET minutes ≤ 1500 min/week). The demographic characteristics of the sample are presented in Table 1.

**Figure 2:**
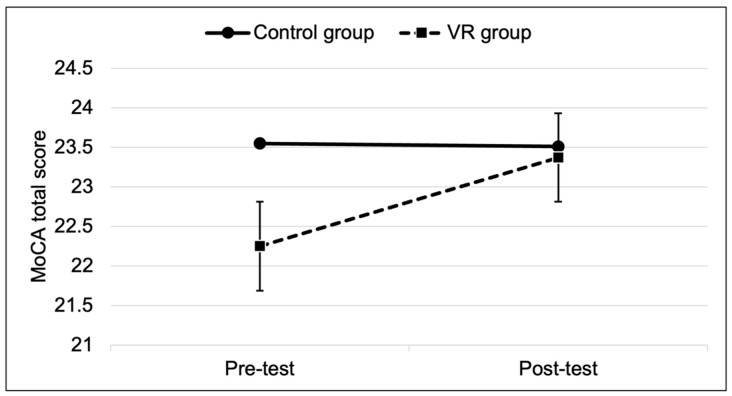
The MoCA total scores (pre- and post-test) for the control and VR groups.

The results indicated that there was a significant difference in the VR group between the MoCA total score pre-test (mean = 22.3, SD = 2.25) and the MoCA total score post-test (mean = 23.4, SD = 2.48), t (23) = 1.87, *p* = 0.03. There was no statistically significant difference in the control group between the MoCA total score pre-test (mean = 23.6, SD = 2.80) and the MoCA total score post-test (mean = 23.5, SD = 3.09), t (19) = 0.08, *p* = 0.46. Figure 2 presents the differences in the MoCA total scores for the control and VR groups.

In order to evaluate multiple domains of the MoCA in both groups, a paired-sample *t*-test was conducted to evaluate the difference in the MoCA score of each domain separately for the control group and VR group. Table 2 summarizes the differences observed for each domain.

To evaluate the PA intensity during the VR game, the MET was calculated using the ActivPAL accelerometer and compared with that of the control group. In addition to the ActivPAL MET, the total number of steps, total time of stepping, number of sit-to-stands, and number of stand-to-sits were identified. Due to the nature of the experiment, there were significant differences between the control group and VR group in these variables, as presented in Table 3. The PA intensity generated from the VR game was equivalent to MVPA, with a mean of 4.98 METs (SD = 1.20).

## 4. Discussion

The current study evaluated the cognitive abilities of 44 sedentary female university students before and after playing an interactive VR game. According to the findings, the cognitive abilities of the VR group were improved compared to the control group, indicating that VR games positively influence cognitive performance. In the trial, the VR game influenced naming, abstraction, and orientation from the MoCA scale most significantly, indicating the significant effects of VR on these domains. According to the METs, the VR game generated a PA intensity equivalent to MVPA in the sedentary university students, which indicates that the VR game has the potential to enhance an individual’s PA and replace traditional exercise methods. As a result of these findings, PA is further supported as an effective tool to enhance general cognition [46,47,48]. Through the physiological changes that occur during exercise, aerobic fitness is expected to enhance cognitive performance through an increase in neurotransmitter availability, improved blood flow to the brain, and enhancements in physiological and neurological processes [47].

Since female students tend to be less physically active than male students, the purpose of this study was to promote female students’ participation in physical activity to minimize the adverse effects of inactivity on their health. A lack of adherence is a major barrier in encouraging an active lifestyle among this age group as they transition into adulthood and experience rapid lifestyle changes. An analysis of existing longitudinal studies has revealed a decrease in PA during the transition from adolescence to adulthood. According to the findings of the 49 qualifying studies, the daily MVPA decreased by 5.2 min per day on average over a period of 3.4 years [67]. Hence, it is important to find alternative means of exercising, such as playing VR games, which influence MVPA, maintain engagement, and ensure adherence. This study provides evidence that VR games enable MVPA in female university students, thus facilitating PA participation and meeting the recommendations of national and international guidelines.

In various clinical populations, VR games have been shown to be beneficial in improving cognitive performance [68,69,70,71]. Studies have shown that VR games improve cognitive skills, memory, and overall cognitive function in a variety of conditions, including Alzheimer’s disease, dementia, brain tumors, and cognitive impairments [72,73,74]. Previous research has indicated that AVGs may provide users with a more exciting and motivating exercise experience than traditional methods [75,76,77,78]. Additionally, AVGs were helpful to both frequent and infrequent exercisers, emphasizing the effectiveness of exergaming across a wide range of PA levels [35]. Another study, however, compared a VR-based exercise cycling session with a stationary cycling session and found no significant difference in blood pressure (BP). While the traditional cycling exercise resulted in more exertion, it was associated with less enjoyment and self-efficacy than the VR-based exercise [27]. In a similar study, McDonough et al. (2020) found that participants experienced greater changes in their systolic blood pressure during VR cycling than through traditional stationary cycling. However, VR-based cycling had little impact except on self-efficacy and the enjoyment of the experience [79].

An experiment randomized 30 healthy young adults into two groups: one participating in an interactive VR game while cycling and the other cycling without playing a VR game. The Wisconsin Card Sorting Task (WCST) was used to measure cognitive flexibility and the Stroop test was used to evaluate cognitive interference inhibition. Both groups showed improved cognitive flexibility, but only the experimental group showed improved selective attention [80]. Furthermore, a study examining the effects of AVGs on recognition memory in 29 university students was conducted using the Mnemonic Similarity Test (MST). They found that AVGs increased the recognition scores and facilitated MVPA when compared to the control group [81]. In line with our findings, they found that AVGs had a significant impact on cognitive performance. Despite this, they used different cognitive scales, VR games, and exercise intensity measures, so a comparison with their findings should be performed with caution. Although the type of VR game was different, our study also included only one VR game (Beat Saber), which may have been difficult or unfamiliar to the female participants; this may have an effect on the generalizability of this study. In future studies, different VR games could provide a more inclusive experience and help to ensure that the results are representative of a wider range of participants. Additionally, offering a variety of game genres could enhance participants’ engagement and yield more comprehensive data.

To investigate the influence of immersion on cognitive performance and the player experience, an immersive VR exercise game was compared to a non-immersive VR exercise game in a randomized clinical trial. These game conditions did not significantly differ in terms of cognitive performance or arousal between 32 participants (18 to 34 years of age). The VR condition, however, significantly increased players’ sense of presence, motivation, and perceived exertion compared to the non-VR condition [82]. Unlike our findings, the researchers concluded that VR exercise games did not have an impact on cognitive performance, which may be attributed to the wide age gap between the study participants. Another study examined the effects of VR exercise games on mood and executive function in 12 young adults. Three groups (rest, exercise, and VR exercise) were evaluated via the color–word Stroop task and they found that VR exergaming resulted in an enhanced positive mood, enabled MVPA, and increased the arousal levels [83]. However, the lack of mood and arousal data in our study makes it difficult to draw comparisons in these areas. It would be beneficial to integrate these measures into future research as a factor influencing the VR game experience.

We used the MoCA test in the present study because it provides a comprehensive evaluation of important cognitive functions, is valid, and can be completed quickly. Previous studies have used the MoCA to investigate the effects of brain training exercises [84] and PA [85] on cognitive performance in healthy young adults. This study’s findings support the MoCA’s applicability and utility in the context of healthy young adults. This is in line with our own findings indicating that the MoCA can detect changes in cognitive performance in young healthy adults. As compared with other tools, such as the MMSE or the General Memory Index (GMI), the MoCA has been shown to be capable of detecting age-related cognitive changes in young healthy adults. Studies have demonstrated its usefulness across the life spans of adults and in comparison to other clinical populations (such as congenital heart patients) [86]. This makes it an ideal tool for the screening and monitoring of cognitive performance in young healthy adults, as well as providing valuable insights into the effects of ageing and disease.

Although this study focused on one gender and had a larger sample size than previously published studies, it has a number of limitations that should be addressed. First, a stricter methodology would have prevented the high number of participants excluded due to device malfunctions and no shows. We were unable to assess the exercise intensity accurately because we did not have a variety of exercise intensity measurements, such as the heart rate, blood pressure, and respiratory rate. Therefore, future research should take this into account. Furthermore, in comparison with cross-sectional studies of a larger scale, RCTs typically recruit a smaller sample size; thus, caution should be exercised when comparing our findings to those of other studies, especially with those of a non-RCT design. This study measured the short-term effect of VR games on cognitive function; however, the long-term and cumulative effects of this intervention should be taken into consideration when designing future studies. Lastly, limiting the cognitive performance measure to a pen-and-pencil scale (i.e., MOCA) may make it difficult to capture other variables, such as reaction times, which may be captured by computerized measures. Additional cognitive measures could be applied in future research to provide a more comprehensive assessment of cognitive function.

## 5. Conclusions

The current study examined the cognitive performance of sedentary female university students and found that an AVG played a short-term positive role in this group’s cognitive performance. Active video games may enable MVPA and, therefore, increase the PA levels and reduce inactivity among sedentary young female. An increase in female young adults’ PA would likely contribute to their academic success, general health, and mental well-being.

## Figures and Tables

**Table 1:** Demographic characteristics of the included sample.

Variable	Control Group	VR Group	*p*-value
Age, years	21.65 (1.08)	21.16 (1.12)	0.15
Weight, kg	59.60 (10.37)	55.20 (12.76)	0.22
Height, cm	156.55 (4.83)	159.20 (5.55)	0.10
BMI, kg/m^2^	24.29 (3.96)	21.72 (4.58)	0.05
Preferred leg, n (%)			
Right	20 (100)	24 (100)	1.0
MoCA pre-test	23.55 (2.80)	22.25 (2.25)	0.09
MoCA post-test	23.50 (3.08)	23.37 (2.48)	0.88
IPAQ-SF weekly MET (min)	525 (479)	609 (797)	0.68

Independent-sample *t*-test, data presented as mean (SD) or number (percentage); VR: virtual reality; kg: kilogram; cm: centimeter; BMI: body mass index; MoCA: Montreal Cognitive Assessment; IPAQ-SF: International Physical Activity Questionnaire—Short Form; MET: metabolic equivalent of task; min: minutes.

**Table 2:** MoCA scores for control and VR groups in pre- and post-test.

MoCA Domain	Control Group		d	VR Group		d
	Pre-Test	Post-Test		Pre-Test	Post-Test	
Visuospatial/Executive	3.65 (0.87)	3.65 (1.04)	0.00	3.67 (1.01)	3.42 (1.06)	0.21
Naming	2.95 (0.22)	3.0 (0)	0.22	2.87 (0.33)	3.0 (0)	0.37 *
Attention A	1.80 (0.41)	1.65 (0.48)	0.25	1.58 (0.65)	1.79 (0.50)	0.23
Attention B	1.0 (0)	0.95 (0.22)	0.22	1.0 (0)	1.0 (0)	0.00
Attention C	2.15 (0.98)	1.85 (1.22)	0.32 *	1.83 (1.0)	2.0 (1.06)	0.16
Language A	1.30 (0.57)	1.20 (0.61)	0.12	1.45 (0.58)	1.66 (0.48)	0.25
Language B	0.10 (0.30)	0.30 (0.47)	0.48 *	0.08 (0.28)	0.20 (0.41)	0.27
Abstraction	1.50 (0.68)	1.35 (0.74)	0.20	1.70 (0.46)	1.41 (0.50)	0.46 *
Delayed recall	3.30 (1.34)	3.65 (1.42)	0.24	2.29 (1.51)	2.95 (1.51)	0.28
Orientation	5.80 (0.41)	5.90 (0.30)	0.32	5.75 (0.44)	5.91 (0.28)	0.43 *
Total score	23.55 (2.80)	23.50 (3.08)	0.01	22.25 (2.25)	23.37 (2.48)	0.38 *

* Significance level *p* < 0.05; paired-sample *t*-test; data presented as mean (SD); VR: virtual reality; MoCA: Montreal Cognitive Assessment; d: Cohen’s d.

**Table 3:** ActivPAL variables calculated in control and VR groups.

Variable	Control Group	VR Group	t	d
METs using ActivPAL	0.02 (0.01)	4.98 (1.20)	18.48 *	5.60
Total number of steps	3.01 (0.01)	146.92 (46.34)	14.15 *	4.28
Total time of stepping (min)	0.02 (0.01)	5.47 (1.93)	12.67 *	3.83
Number of sit-to-stands	0.01 (0.01)	2.13 (1.08)	8.82 *	2.67
Number of stand-to-sits	0.02 (0.01)	2.50 (1.22)	9.18 *	2.78

* Significance level *p* < 0.001; independent-sample *t*-test, data presented as mean (SD); VR: virtual reality; MET: metabolic equivalent of task; min: minutes; d: Cohen’s d.

## Data Availability

The datasets used and/or analyzed during the current study are available from the corresponding author on reasonable request.
